# Abnormal Static Sagittal Cervical Curvatures following Motor Vehicle Collisions: A Retrospective Case Series of 41 Patients before and after a Crash Exposure

**DOI:** 10.3390/diagnostics14090957

**Published:** 2024-05-02

**Authors:** Jason W. Haas, Paul A. Oakley, Joseph R. Ferrantelli, Evan A. Katz, Ibrahim M. Moustafa, Deed E. Harrison

**Affiliations:** 1 Chiropractic Biophysics NonProfit, Inc., Eagle, ID 83616, USA; 2 Kinesiology and Health Science, York University, Toronto, ON M3J1P3, Canada; docoakley.icc@gmail.com; 3 PostureCo, Inc., Trinity, FL 34655, USA; 4 Independent Researcher, Boulder, CO 80302, USA; 5 Department of Physiotherapy, College of Health Sciences, University of Sharjah, Sharjah 27272, United Arab Emirates; 6 Neuromusculoskeletal Rehabilitation Research Group, RIMHS–Research Institute of Medical and Health Sciences, University of Sharjah, Sharjah 27272, United Arab Emirates

**Keywords:** neck pain, cervical lateral radiograph, motor vehicle collision, cervical lordosis, buckling, trauma

## Abstract

Previous investigations have found a correlation between abnormal curvatures and a variety of patient complaints such as cervical pain and disability. However, no study has shown that loss of the cervical curve is a direct result of exposure to a motor vehicle collision (MVC). This investigation presents a retrospective consecutive case series of patients with both a pre-injury cervical lateral radiograph (CLR) and a post-injury CLR after exposure to an MVC. Computer analysis of digitized vertebral body corners on CLRs was performed to investigate the possible alterations in the geometric alignment of the sagittal cervical curve. Methods: Three spine clinic records were reviewed over a 2-year period, looking for patients where both an initial lateral cervical X-ray and an examination were performed prior to the patient being exposed to a MVC; afterwards, an additional exam and radiographic analysis were obtained. A total of 41 patients met the inclusion criteria. Examination records of pain intensity on numerical pain rating scores (NPRS) and neck disability index (NDI), if available, were analyzed. The CLRs were digitized and modeled in the sagittal plane using curve fitting and the least squares error approach. Radiographic variables included total cervical curve (ARA C2–C7), Chamberlain’s line to horizontal (skull flexion), horizontal translation of C2 relative to C7, segmental translations (retrolisthesis and anterolisthesis), and circular modelling radii. Results: There were 15 males and 26 females with an age range of 8–65 years. Most participants were drivers (28) involved in rear-end impacts (30). The pre-injury NPRS was 2.7 while the post injury was 5.0; *p* < 0.001. The NDI was available on 24/41 (58.5%) patients and increased after the MVC from 15.7% to 32.8%, *p* < 0.001. An altered cervical curvature was identified following exposure to MVC, characterized by an increase in the mean radius of curvature (265.5 vs. 555.5, *p* < 0.001) and an approximate 8° reduction of lordosis from C2–C7; *p* < 0.001. The mid-cervical spine (C3–C5) showed the greatest curve reduction with an averaged localized mild kyphosis at these levels. Four participants (10%) developed segmental translations that were just below the threshold of instability, segmental translations < 3.5 mm. Conclusions: The post-exposure MVC cervical curvature was characterized by an increase in radius of curvature, an approximate 8° reduction in C2–C7 lordosis, a mild kyphosis of the mid-cervical spine, and a slight increase in anterior translation of C2–C7 sagittal balance. The modelling result indicates that the post-MVC cervical sagittal alignment approximates a second-order buckling alignment, indicating a significant alteration in curve geometry. Future biomechanics experiments and clinical investigations are needed to confirm these findings.

## 1. Introduction

Neck strain/sprain is the most common type of diagnosis given to motor vehicle collision (MVC) occupants treated in United States hospital emergency departments [1]. An anatomical non-systematic review by Curatolo et al. found tissue damage from collisions to the “zygopophysical joint capsules, annuli fibrosi; partial or complete ruptures of capsules, ligaments, annuli fibrosi intra-articular contusions, intra-articular fractures; and transarticular synovial joint fractures” [2]. Conditions such as headache, neck pain, and myelopathy with and without neuropathic radiculopathies resulting from MVC’s frequently become chronic and debilitating. Studies have reported that between 17% and 45% of injured persons have lingering dysfunction, pain, and disability at long-term follow-up [2,3,4,5,6,7,8,9,10,11,12,13,14].

In patients with injuries resulting from an MVC, a correlation between S-shaped, kyphotic, or hypo-lordotic cervical curve configurations and cervicogenic pain, radiculopathies, and increased rates in degenerative discs have been reported [15,16,17,18,19,20,21,22,23]. These abnormal structural configurations are associated with multiple symptoms and worsening of prognosis [15,16,17,18,19,20,21,22,23]. According to Nightingale et al. [24], referring to Chen and Lui [25], “In a column with a fixed base, buckling is evidenced by an abrupt decrease in measured compressive load with increasing deflection and moment. Snap through buckling is characterized by a visible and rapid transition from one equilibrium configuration to another.” Biomechanical investigations confirm that the sagittal cervical spine exhibits ‘snap-through’ type and ‘dynamic’ buckling behavior in response to impact loads, where complexity is related to curve configuration, posture at impact, rate, and magnitude of loads [24,26,27,28].

In MVC’s acceleration/deceleration events, during the skull translation phase, many authors have described an S-Curve (first-order buckled mode) of the cervical lordosis [29,30,31,32,33]. Furthermore, altered geometry of the static cervical curvatures of patients has been previously described as buckled modes/shapes [34], but to our knowledge no study has shown that MVC’s cause or contribute to altered geometry of the cervical curve post-crash. Since no study has conclusively demonstrated that the MVC itself is a causation of an abnormal static cervical lordotic alignment, we wished to investigate this possibility. Using a retrospective consecutive case series design, 41 patients were obtained where a pre-injury cervical examination and X-ray were followed by a second cervical examination and X-ray after exposure to an MVC. The current study’s hypothesis is that MVC exposure will cause a buckling type of altered cervical spine alignment segmentally and globally in the sagittal cervical spine.

## 2. Materials and Methods

### 2.1. Patient Data Collection

For the current investigation, we completed a 3 center retrospective consecutive case series review of records over a 2 year time period. This article is a retrospective review of clinical records, and is exempt from IRB approval under Section 45 CFR 46.101(b)(4). See: https://www.hhs.gov/ohrp/regulations-and-policy/decision-charts-pre-2018/index.html#c5 (accessed on 13 February 2024). Patients were included where an initial neutral lateral cervical radiograph and cervical spine examination was performed prior to any patient being exposed to the MVC. Prior to exposure to the MVC, all patients were seeking care for cervical spine complaints (unrelated to any MVC) and were under the care of their primary care Chiropractic physicians in each of these three private practices. Following their initial course of treatment for non-MVC-related cervical spine conditions, this included a sample of patients who were involved in an MVC. After exposure to this MVC, each patient received a repeated examination and second cervical radiograph as part of a routine screening for cervical spine trauma. Thus, the patients returned to their same provider (the same Chiropractic center) for a second examination and radiograph after being exposed to the MVC. Thus, both initial (pre-MVC) and follow-up (post-MVC) exams and radiographs were performed at the same location in order for fidelity. The post-MVC exposure patient examination and radiographs were included if the patients had at least 24 h and up to 6 weeks following the time of their MVC exposure and injury.

In addition to lateral cervical radiographs, patient examination records were required to have completed the numerical pain rating score (NPRS) from 0–10 (where 0 = no pain and impairment, 10 = severe incapacitating pain) at both examinations. The reliability [35] and validity [36] of the NPRS is excellent for clinical usage. Many patients also completed the neck disability index (NDI) to assess the impact of their complaints on their activities of daily living. The NDI is reliable, valid, and has good responsiveness to change [37].

Exclusion criteria for patients in our study were: (1) pre-existing and post-MVC cervical spine fractures, (2) pre-existing cervical spine instability, (3) pre-existing cervical spine surgery, (4) non-ambulatory or unable to maintain a neutral cervical posture, (5) duration between examinations of more than 1 year, (6) failure to present to the same clinic as the primary center within 6 weeks after the MVC, and (7) patients with sensory or motor deficits consistent with moderate-severe radiculopathy due to disc herniation and/or myelopathy cases. Further, there were no patients with dislocation, fracture of the pedicle, facet, lamina, or other bony structures in our population after exposure to the MVC. Forty-one patients were identified that fit our inclusion criteria. These persons ranged in age from 8–65 years, and there were 15 males and 26 females.

### 2.2. Cervical Circular and Elliptical Modeling

All 82 (2 sets of 41 patients) lateral cervical spine radiographs were digitized with a sonic digitizer (GP-9, from Science Accessories Corp., Shelton, CT, USA). Digitized points included: (1) the posterior hard palate, (2) the posterior portion of the foramen magnum, (3) the anterior and posterior tubercle of the atlas, and (4) all 4 vertebral body corners of C2–C7. In total, 32 combined points on the skull and cervical vertebral bodies from C1–C7 were digitized. The digitization and modeling details have been previously reported; however, some details are provided here [38].

Our circular and elliptical models extend from the posterior–superior body corner of C2 to the posterior–inferior corner of the body of C7 or to T1; the model represents the path of the posterior vertebral body coordinates between these landmarks [38]. Vertebral body x–y coordinates were stored in a database for a computer program written in FORTRAN 77 to run on a personal computer. An original computer code performed a least squares approximation of each person’s cervical lordosis in the shape of an ellipse or a circle. The program iterates to find a best-fit model for each person by passing ellipses and circles, in the least-squares sense, through vertebral x–y coordinates along the posterior bodies. For each person’s best-fit ellipse, the program determined semi-major (a) and semi-minor (b) axes, b/a ratio, the portion of a quadrant (between 80° and 90°), which the elliptical segment comprised; and for circular models, the radii of curvature is determined [38].

### 2.3. Radiographic Procedures and Variables

All three clinics followed the same standardized procedure for patient positioning during the exposure of the lateral cervical radiographic [38,39]. Each patient stood next to the X-ray bucky with the shoulder touching lightly. The tube source was 183 cm (72 inches) away from the bucky, and the collimation allowed the entire cervical spine to be visualized. The patient was instructed to gently nod their head a few times with their eyes closed and to assume a neutral “eyes-forward” position with their eyes open to prevent sway. Once the patient was still, the image was acquired. This positioning has been shown to be repeatable and reliable [39].

#### Cervical Spine Radiographic Measurement Variables

**Figure 1:**
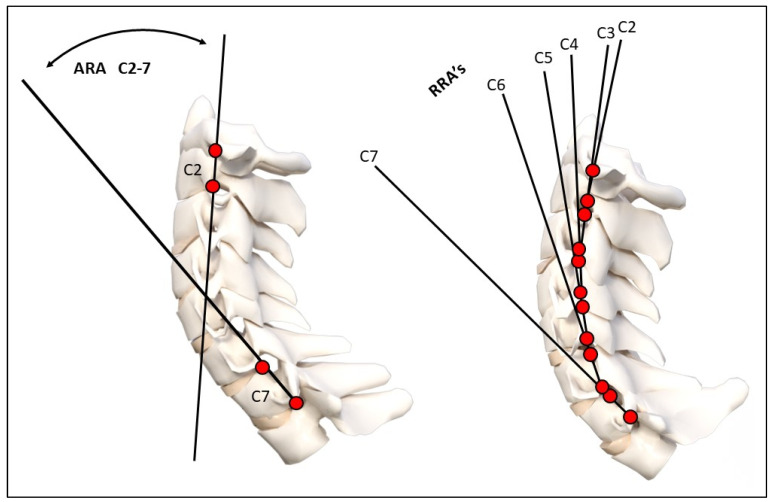

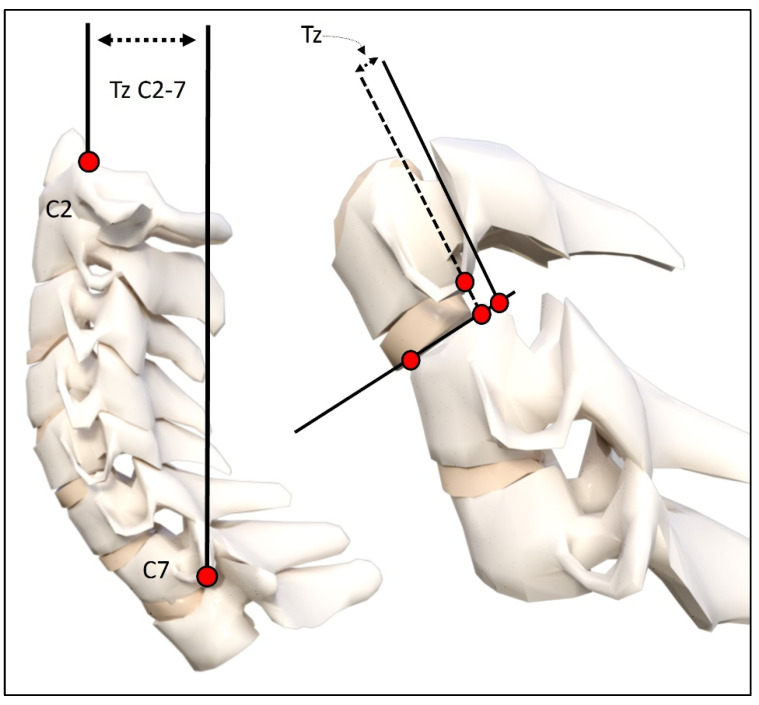
Radiographic mensuration techniques evaluated. Top image: Harrison posterior tangent method analysis of the cervical lordosis ARA C2–C7 and RRAs (note: RRA’s were represented using curve fitting and circular modelling). Bottom image: horizontal translation of C2–C7 and intersegmental translation between individual vertebrae. ARA: absolute rotation angle, RRA: relative rotation angle. These measurements have excellent examiner reliability and small examiner error magnitudes [40,41].

Using the digitized vertebral body x–y coordinates, sagittal cervical angular (rotations) and linear (translation) alignment variables were calculated. These variables included: (1) the intersection of posterior body lines at C2 and C7 forming a global angle of lordosis (ARA C2–C7), (2) intersection of juxtaposition posterior body lines forming relative rotation angles (RRA’s) from C2–C3 down to C6–C7, (3) a line through the posterior hard palate and the posterior foramen magnum relative to horizontal (Chamberlain’s line), (4) horizontal translation of the posterior superior vertebral body corner of C2 relative to a vertical line at the posterior inferior vertebral body corner of C7 (Tz C2–C7), and (5) segmental forward and backward translations of juxtapositioned vertebra (retrolisthesis and anterolisthesis) [40,41]. See Figure 1. A negative angular value indicates spinal extension or lordosis, and a positive linear value indicates anterior translation. These measurements have excellent examiner reliability and small error magnitudes [40,41].

### 2.4. Statistical Analysis

Descriptive statistics are presented as mean and standard deviations for all variables. Statistical comparisons of the changes in cervical modeling, radiographic alignment variables, pain, and disability scores following exposure to an MVC were performed using a two-tailed paired observations *t*-test. In order to determine the sample size needed for statistically significant data, we calculated Cohen’s d effect size for the known change in our patients’ ARA C2–C7 lordotic angle following their MVC (where d = 0.581). Using this information, we calculated a sample size needed of *n* = 20 with a statistical power of 0.8 and a probability level of 0.05. Lastly, we performed an assessment of the potential effects of age and sex using a two-sample *t*-test to compare the ARA C2–C7 difference from pre- to post-collision between males and females and those over 16 and 16 years and under. Patient data were initially imported into Microsoft Excel (2018 Microsoft Excel (office.microsoft.com/excel, accessed on 13 February 2024)), and statistical analyses were performed with SPSS version 29.

## 3. Results

### 3.1. Patient Demographics

Patient demographics, passenger vehicle location, type of collisions, NPRS, and NDI scores are presented in Table 1. The majority of patients were in the front driver location (28/41) of the target vehicle involved in a rear-end collision (30/41). On average, patients’ pain intensity nearly doubled from an NRS of 2.7 to a 5.0 following exposure to an MVC; *p* < 0.001. Similarly, the NDI was available on a subset of 24/41 (58.5%) patients, and it was also found to increase by a factor of two following exposure to the MVC (15.7% to 32.8%), indicating a change from mild to moderate impairment on the NDI.

### 3.2. Change in Cervical Curvature from MVC

**Figure 2:**
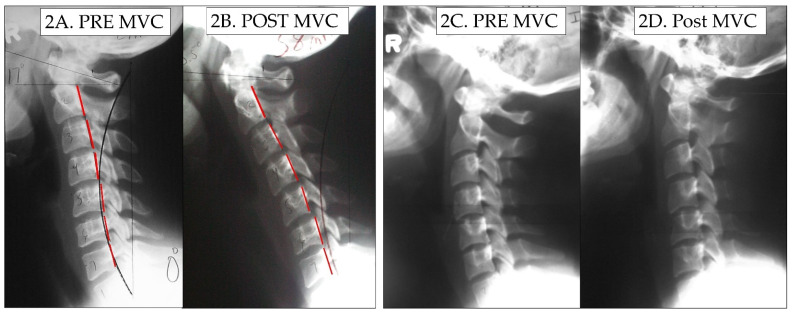
Before and after MVC alteration in the cervical lordosis. In (**2A**), a female patient’s initial cervical curve, and in (**2B**), the post-MVC cervical curve is shown to be significantly altered. In (**2C**), a second female patient’s initial cervical lordosis, and in (**2D**), the same patient’s post-MVC lateral cervical radiograph is shown with a significant reduction in lordosis, especially throughout the mid-cervical segments. The red dashed line represents the path of the patient’s posterior vertebral body margins in the sagittal plane from C2–C7.

The means, standard deviations, maximum, and minimum values for the measured radiographic variables in the 41 patients are reported in Table 2. There were nine patients who were aged 16 years and under. There was not a significant difference in those over 16 years of age for the change in ARA C2–C7 cervical lordosis (6.8 ± 9.1°) compared to those under 16 years of age (11.6 ± 9.1°); t (39) = 1.272, *p* = 0.237. We assessed the potential effects of sex on the change in the ARA C2–C7 from pre- to post-collision between males and females, and there was no significant difference between males (5.0 ± 8.6°) and females (9.1 ± 9.3°); t (39) = −1.434, *p* = 0.161. Thus, Table 2 presents the combined data of all 41 patients as a group. An altered cervical curvature was identified following exposure to MVC, and was characterized by an increase in radius of circular modelling curvature, an 8° reduction (range of 40.7° loss to 5° increase) in the ARA C2–C7, and straightening/flexion of the mid-cervical spine from C3–C5 (6.5° loss). In 3/41 participants a mild increase in the ARA C2–C7 was found; however, the geometry was significantly altered with an increase in the radii of curvature. Table 2 presents these data. Figure 2 shows two female patients where the cervical curve is demonstrably altered from post-MVC exposure compared to their initial lateral cervical radiographs.

### 3.3. Modeling Results

**Figure 3:**
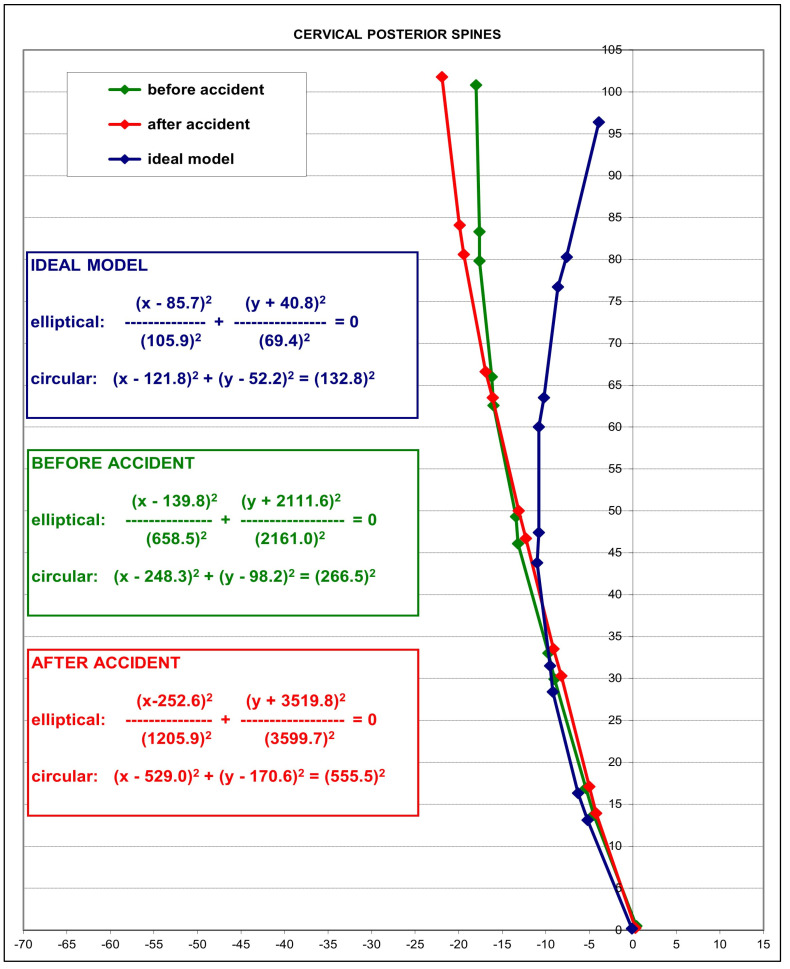
Modeling results. In blue, an average idealized model of cervical lordosis is shown [38]. In green, the pre-MVC patient average model is shown. In red, the post-MVC patient model is shown. Modelling methods are described in detail in the report previously presented [38].

**Figure 4:**
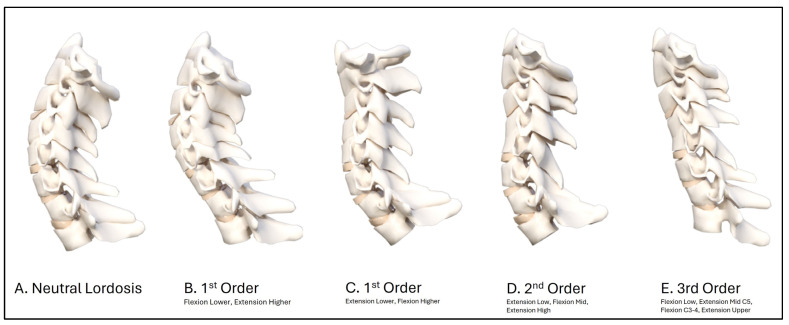
A selection of possible buckled modes for the sagittal cervical during and after exposure to an impact or inertial loading event such as a motor vehicle crash collision. Increasing complexity is shown by increasing the number of directional or slope changes. (**A**) The normal neutral cervical lordosis is shown. (**B**) A 1st order buckled mode is shown with flexion in the lower cervical and extension in the upper cervical spine. (**C**) An opposite first-order buckled mode in comparison to B where there is extension in the lower cervical and flexion in the upper cervical spine. (**D**) A second-order buckled mode is shown with an extension in the lower cervical, a flexion in the mid cervical, and an extension in the upper cervical region. (**E**) A third-order buckled mode is shown with a flexion in the lower, an extension at C5, a flexion at C3–C4, and an extension at C0–C2.

In 2004, an average model as a piece of a circle with an angle of 34.5° between the posterior body tangents on C2 and C7 was reported [38]. This model is shown blue in Figure 3 as a representative ‘idealized aligned model’, and is compared to the initial patient average (green model) and post-MVC exposure (red model) average cervical model of the 41 participants. The averaged post-MVC lateral cervical curve model approaches a second-order buckled mode with straightening/flexion of the mid-cervical segments C4-C6 compared to the pre-injury model of the lateral cervical radiographs. The altered geometry is shown by the change in equation values and specifically by the increase in the radius of curvature on the before-MVC vs. the after-MVC model (radii of curve change from 266.5 mm^2^ to 555.5 mm^2^). Furthermore, the increase in anterior translation of C2–C7 is shown in the post-MVC model in Figure 3. Figure 4 depicts a representative sample of possible buckled modes in the sagittal cervical spine. During impact experiments, the cervical spine has been found to exhibit “snap-through” and dynamic-type buckling behavior [24,26,27,28,29]. Note, our post-MVC model in Figure 3 approximates a second-order buckling, as shown below in Figure 4D.

**Figure 5:**
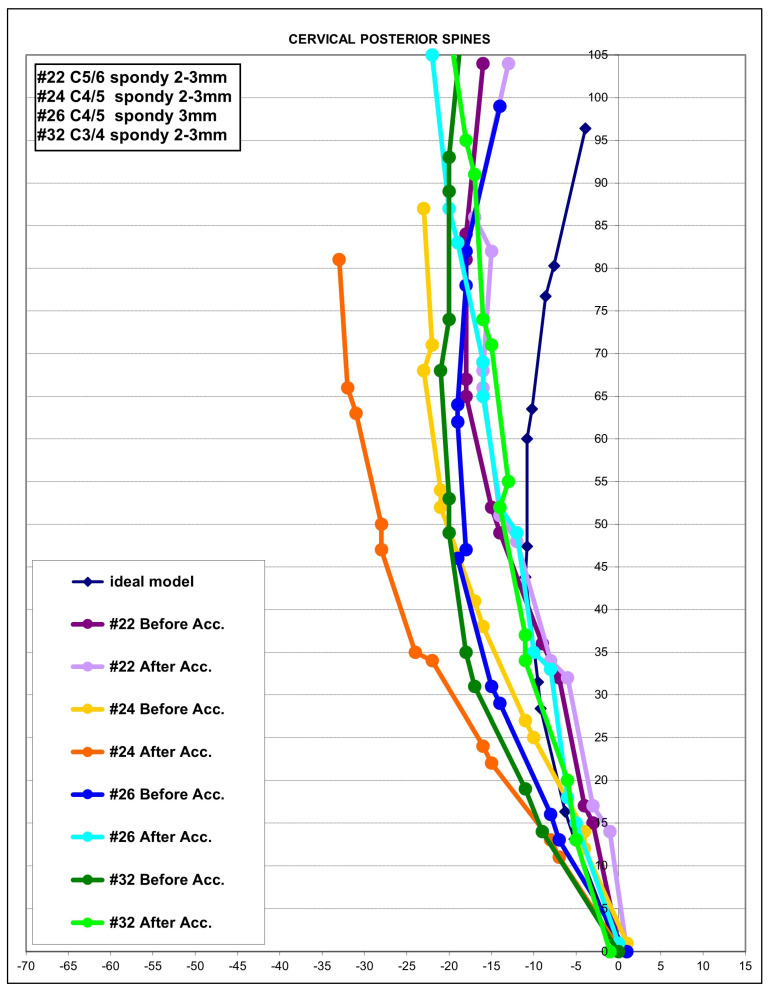
Segmental translation modeling results. In 4/41 patients (10%), segmental translations were identified on the post-MVC radiographs that were different than the ones identified on the initial pre-MVC radiograph. Each of these patient’s model is shown pre- and post-MVC as a change in color and coded by their number out of 41. Acc.: = MVC. # represents the patient number in the sample of 41 patients. # represents the patient number in the sample of 41 patients.

In 4/41 (10%) patients, significant segmental translations (retrolisthesis and anterolisthesis) on the post-exposure MVC lateral cervical radiograph were found that were quite different in comparison to the initial pre-MVC lateral cervical radiograph. The magnitude of these segmental translations approached the limits of allowable maximum joint movement (3.5 mm) before instability occurs, and represents abnormal alignment suggestive of ligamentous injury. Note that this threshold does not exceed the limit of true translation instability, and thus is consistent with sub-catastrophic (micro) damage to the connective tissues of the cervical spine [23,33]. Figure 5 presents the segmental translation of the individual segments identified on the post-MVC radiographs of these four patients. Each person’s model is shown pre- and post-MVC.

## 4. Discussion

The present study was undertaken to compare the sagittal cervical spine geometry and radiographic alignment within patients before and after exposure to a motor vehicle collision (MVC). We had hypothesized that following exposure to an MVC, a patient’s cervical lordotic curvature would be altered as a result of the crash. The cervical model and radiographic alignment variables before and after MVC exposure confirm our primary hypothesis that MVC causes a buckling type of alignment in the sagittal cervical spine that is noted both segmentally and globally. In our retrospectively analysis of 41 participants, a general hypo-lordotic cervical curvature was identified following exposure to an MVC and was characterized by an increase in radius of curvature, an approximate 8° reduction (up to 40.7° in one patient) in the ARA C2–C7, and mild kyphosis of the mid-cervical spine from C3–C5 (6.5° loss). Finally, a mild increase in forward head posture was found following exposure to an MVC, as measured with the C2–C7 translation distance. To our knowledge, our investigation is the first study we know to report abnormal cervical curvature within a group of patients following exposure to an MVC. However, our findings seem consistent with the general radiographic findings in MVC-injured persons compared to matched control groups [17,18,19,20,21,22,23,42].

### 4.1. Loss of Lordosis in MVC Populations

Taken as a whole, the literature detailing patients involved in a MVC and those with whiplash-associated disorders (WAD) indicates that hypolordotic [16,17], straightened cervical curves [16,17,18,19], S-curves [18,21,23], and kyphotic curves [15,18,19,20,21] are risk factors for, and are statistically correlated to, generalized poor long-term outcomes including chronic neck pain. Data from Marshall [42], for example, provide evidence that patients involved in an MVC injury have a 10° mean reduction in the C1–C7 Cobb angle of cervical lordosis compared to a control group. Complicating matters is that conflicting results have been reported whereby the relationship of altered cervical curvatures to prognostic outcomes following exposure to an MVC (both short- and long-term) has been refuted [43,44,45,46]. Still, according to our review, the majority of the literature indicates that patients with injuries resulting from an MVC have a significantly greater frequency and magnitude of altered cervical curve configurations [15,16,17,18,19,20,21,23,42]. This information implies one of two things: (1) that either patients presenting with a pre-existing abnormal cervical spine curvature are predisposed to injury of their cervical spine tissues from the MVC event, or (2) that the MVC event causes a mechanical alteration (buckling event) in the alignment of the lordotic cervical alignment, leading to increased risk of injury and future pain and disability. In fact, the first of these scenarios has been preliminarily reported in the literature [47,48,49], and the results of the current investigation indicate that the second scenario also holds true.

There have been numerous studies demonstrating the chronic effect of abnormalities of the cervical lordosis both in cervical spine trauma scenarios and degenerative spine disorders. The consequences for long-term increases in pain, loss of function, and increased risk for disability have been detailed in the surgical [50], MVC [18,19,20,21], and conservative care rehabilitation literature [51,52]. Further, studies have shown that both non-surgical and surgical improvement in cervical lordosis is a desirable clinical goal of care, with significant short- and long-term improved outcomes [50,51,52]. Problematically, we could locate no randomized trial demonstrating the long-term superiority of results for cervical lordotic rehabilitative correction groups in patients suffering from MVCs, and this represents an important area of future research.

### 4.2. Altered Sagittal Cervical Geometry as a Buckling Type Alignment

An S-shape in the cervical column can be described as the first-order buckled mode, flexion-extension-flexion in any region as the second-order buckled mode, etc., where these are increasing in complexity based on the number of directional or slope changes in the sagittal cervical spine. Buckling can be segmental rotations or translations. Figure 4 depicts a representative sample of possible buckled modes in the sagittal cervical spine. During impact experiments, the cervical spine has been found to exhibit “snap-through” and dynamic-type buckling behavior [24,26,27,28,29]. The various buckled modes are the allowable shapes that the sagittal cervical curve can adopt without failure, and these can be correlated to eigenvalues in solutions of nonlinear partial differential equations used to model structures [25,53,54]. During exposure to an MVC, the cervical spine is subjected to complex inertial loading where bending, shear, and compression loads act on the column. During the skull translation phase, many authors have described an S-Curve (first-order buckled mode) of the cervical lordosis [29,30,31,32,33]. Similarly, Matsunaga et al. [34] described altered static cervical curvatures, including S-curves and retrolistheses, as buckled modes or shapes. The cervical spine exhibits “snap-through” and/or dynamic buckling behavior in some cases when exposed to high inertial loading, as in cervical acceleration–deceleration. We believe this to be a possible explanation as to why the average post-MVC alignment of our population is consistent with a second-order buckling alignment (extension C6-T1, flexion C4-C6, and extension C1-C3), as shown in Figure 3, and why some of our patients’ curves developed higher-order buckling (third-order or greater, as shown in selected cases in Figure 5). Our results are in agreement with those of Kristjansson and colleagues [21], who identified that females involved in an MVC had a decreased ratio of the upper vs. lower cervical lordosis with a flexion at the C4/C5 segmental level.

One of the most common hypotheses regarding loss of the cervical lordosis following exposure to an MVC is that cervical kyphosis or hypolordosis is due to muscular spasms in the cervical region. The evidentiary support for this assertion is based on class V (expert opinion) evidence exclusively [55,56,57,58]. In contrast, as early as the 1960s, Rechtman et al. [59] stated, “*Flattening of a cervical lordosis should be evaluated, carefully, especially in medicolegal problems, before being attributed to muscular spasm, as has been mentioned so commonly in radiologic reports. The muscular response associated with loss of cervical lordosis remains for further clarification.*” Helliwell et al. [60] found no relationship between muscle spasm and loss of cervical lordosis. They [60] suggested that muscle spasm would cause an increased lordosis due to the larger volume and larger moment arms of the posterior extensor muscles of the cervical spine. Lastly, Fedorchuk et al. [61] found no evidence that hypertonicity and contraction of the cervical spine musculature has a significant impact on shape and magnitude of the cervical lordosis. According to the above review, there is no scientific data to date proving that loss, reversal, or buckled alignments of the cervical curve merely reflects muscle spasm in the cervical region. Importantly, cervical spine snap-through buckling occurs 2–3 times faster than the muscles can fully react to an MVC collision event [47,48,49,62]. Therefore, we argue that cervical spine muscle spasm or hyper-tonicity is a co-variable of the reaction to an injury, and is not a cause of altered cervical sagittal alignment.

### 4.3. Pain Increase Post-MVC and Clinical Consideration

In our population, following exposure to a MVC, the average NRS pain score and neck disability index were found to nearly double, being reported as a 5/10 for the NRS.

All of our 41 patients were initially seeking treatment for cervical spine complaints prior to their exposure to an MVC, and cervical spine radiographs were acquired as part of their routine examination at the representing spine treatment centers. It is noteworthy that their initial lateral cervical lordotic alignment averaged 17° from C2–C7 (Table 2), and this is less than the 20° threshold that is known to be a statistically significant cutoff value of lordosis for the discrimination of neck pain versus non-neck pain populations; this is true even for children as young as 9 years of age [38,63]. Thus, our patient population, on average, already had a significant reduction in their cervical lordosis prior to their crash, which likely contributed to some of their initial pain findings. Interestingly, investigations have found that pain intensity scores following an MVC are predictive of long-term outcomes in patients with acute whiplash-associated disorders [2,3,4,6]. A pain score of 5/10 on a NRS is considered moderate pain associated with significant disability [2,64]. The significance of this increase in pain cannot be understated in our population of patients. Neck pain is one of the largest contributors to musculoskeletal global burden of disease [64,65,66]. The failure to properly diagnosis neck pain and the parameters of injury can lead to increased risk of chronicity due to increased mechanical stresses and strains on the nociceptive pain-sensitive tissues of the spine. Increased mechanical stress and strain on internal and external spine structures worsen range of motion and strength and worsen outcomes in both the short and long term, including leading to an increased rate of degenerative changes [2,3,4,5,6,7,18,20,66].

Trauma physicians and emergency room (ER) physicians should be aware of these abnormal spine parameters, which are visualized via cervical radiography, prior to considerations for differential diagnosis, and add to the decision for invasive or pharmacologic interventions, determination of referral recommendations for pharmacologic, therapeutic, and/or manual treatments. Surgical referral following stabilization of triage in the ER by the ER physician must have the clinical certainty that the buckling of the cervical spine is significant enough for this type of referral; in severe cases (not included in our population), these can be coupled with cervical fractures and dislocations [47,67]. Conservative, non-invasive, and non-opioid pharmacologic interventions should be considered if possible [68,69]. Proper referral for non-invasive vs. surgical intervention could have a significant impact on the patient outcome, as well as the financial burden of the injury due to the MVC.

### 4.4. Safety of Radiography for MVC Patients

Radiography is the criterion standard for spine conditions. The safety of radiography for injured patients with suspected spine conditions has been firmly established, and the fear of radiography on the part of patients, physicians, and others is not warranted [70,71,72,73]. Additionally, the failure to use radiography to assess the condition of the spine after injury and prior to treatment could lead to poor outcomes, including wrong diagnosis and improper treatment applications, and could increase the likelihood of future pain, dysfunction, and possible escalation of intervention including surgery [67,71,72,73]. Lastly, regarding specific conservative care rehabilitation procedures for MVC-injured patients, recent results demonstrate that the judicious usage of cervical spine radiography aids in the continued management of cervical spine disorders in patients with loss of the cervical curvature resulting from exposure to an MVC [74]. Furthermore, this small treatment case series (*n* = 7) by Norton and colleagues [74] identified that MVCs altered the patient’s post-treatment cervical lordotic alignment by an average of 18.7° (2.5 x the mean reduction reported herein) and that rehabilitation was able to re-establish the altered cervical lordosis; thus, this preliminarily validates the current study’s findings of an altered cervical lordotic curvature following exposure to an MVC.

### 4.5. Study Limitations and Future Recommendations

The current investigation has several limitations inherent in its design. The limitations include problems with retrospective review of records, a time frame of up to a year from the initial examination to the second examination after MVC exposure, and a limited sample size. However, the second examination and lateral cervical radiograph was taken within 1 day to 6 weeks following exposure of the patient to an MVC. The cervical lordosis has been found to be stable in chronic neck pain patients not undergoing treatment intervention for up to 10 years [39,75], and conservative treatments show long-term stability when lordosis is restored [51,52]. Therefore, we doubt that the time between our patients’ first and second exams caused abnormalities on the second lateral cervical radiograph, but we cannot rule out the possibility of other non-reported injuries confounding the outcome. The limited sample size did not allow for adequate correlation between the different impact directions and consequent curve abnormality. Future investigations should compare different collision types and directions of MVCs and compare the post-crash buckling shape, injuries sustained, and presenting symptoms.

Likewise, our sample of patients did not have advanced imaging evaluation with MRI or CT scans of the cervical spine to completely rule out the possibility of more complex soft-tissue and hard-tissue injuries. However, our exclusion criteria eliminated patients with sensory and motor deficits from more advanced radiculopathy, and radiography did not detect these more complex spine injuries. Still, it is unknown if our group of patients was completely free of subtle fractures and more serious ligamentous injuries. It is known that patients with disco-genic radiculopathy following an MVC also have a higher frequency of abnormal alignment of the cervical lordosis compared to a matched control group [17]. Thus, it is possible that this occurred in some of our patients. Finally, treatment studies should investigate the magnitude of the cervical spine ‘buckling’ in relationship to the success of conservative treatments and patient outcome measurement changes with long-term follow-up in order to evaluate whether correction of the altered cervical curve benefits patients undergoing care for MVC-related conditions. Because this study involves injured patients, prospective randomized trials with controls would be unethical and should not be considered; however, large prospective and retrospective studies of patients with spinal radiographs prior to and following an MVC should be funded and undertaken to confirm or refute our investigation’s findings.

A final limitation might be that the repeatability of radiographic positioning procedures to assess the sagittal alignment of the cervical spine could be challenged. However, it is known that the positioning of the cervical spine in the self-balance position or the neutral sagittal posture is highly repeatable within a few degrees from session 1 to session 2 [39]. Therefore, any change in alignment can be contributed to an impact trauma (such as an MVC) or treatment interventions. Furthermore, it might be thought that the mild head flexion difference noted on the lateral cervical image of the patient in Figure 2B compared to their initial pre-injury X-ray in 2A explains the reduction in overall cervical lordosis and the kyphotic configuration. In contrast, the idea that slight head flexion differences (up to 15°) on one film compared to a second film of the same patient causing an alteration in cervical curve geometry has been clearly refuted; slight-to-mild head flexion does not change the magnitude and geometry of the cervical lordosis to this extent [76,77].

## 5. Conclusions

An altered cervical curvature was identified following exposure to a MVC in 41 retrospective cases from three patient centers. The 41 patients had a prior lateral cervical radiograph for comparison to a post-MVC lateral cervical radiograph. The abnormal curvature was characterized by an increase in the radius of curvature, an approximate 8° reduction in C2–C7 lordosis, mild kyphosis of the mid-cervical spine, and a slight increase in the anterior translation of the C2–C7 sagittal balance. Ten percent of the patients developed segmental translations that approached instability limits. Overall, the modelling result of the sagittal cervical lordosis indicates that the post-MVC alignment approximates a second-order buckling alignment, indicating a significant alteration in curve geometry. MVCs would appear to be a causative factor for lateral cervical curve abnormalities, and as such need more attention in terms of research and rehabilitation methods in populations after exposure to an MVC. Future research should include prospective evaluation of curvature change immediately following exposure to an MVC, and future modeling investigations should consider incorporating a post-crash alignment analysis to identify if change in cervical sagittal alignment might be the result of specific patient and crash characteristics. Finally, future randomized trials and case series investigations are needed to explore the results of cervical curve restoration in MVC-injured populations to identify if this may benefit specific subgroups of patients suffering from chronic pain and impairments.

## Figures and Tables

**Table 1:** Patient characteristics, crash direction, pain scores, and radiographic outcomes.

Variable	Mean ± SD	Location in Vehicle	Collision Type	Pre NRS	Post NRS	Cohen’s d for NRS	*p* Value * Change
Height	159 ± 43.5	FD = 28 FP = 10 RP = 3	Rear = 30 Front = 2 Side = 7 Oblique = 2	2.7 ± 2.5	5.0 ± 2.4	0.939	*p* < 0.001
Weight	65.1 ± 4.5			Pre NDI **	Post NDI **	**Cohen’s d** **for NDI**	*p* Value * Change
Sex	M = 15 F = 26			15.7 ± 15.1	32.8 ± 15.4	1.121	*p* < 0.001
Age	33.9 ± 16.9 8–65 years						

Note: M: male; F: female; MVC: motor vehicle crash; NRS: numerical rating scale; NDI: neck disability index; ARA: absolute rotation angle of cervical lordosis; FD: front driver; FP: front passenger; RP: rear passenger. * Two-tailed, paired observations *t*-test; ** NDI data only available on 24/41 patients.

**Table 2:** Means, standard deviations (SD), maximum, and minimum values for all variables used in the analysis of MVC patients.

Variable	Initial Lateral Cervical Radiograph			Post Lateral Cervical Radiograph			Cohen’s d	*p* Value ** Change
	Mean ± SD	Maximum Value	Minimum Value	Mean ± SD	Maximum Value	Minimum Value		
**Tz C2–C7 (mm)**	19.6 ± 9.8	47	2	22.0 ± 12.5	58	−3	0.215	*p* < 0.05
**Chamberlains (°)**	−2.4 ± 6.7	12	−19	−0.96 ± 6.6	14	−12	0.217	*p* < 0.05
**ARA C2–C7 (°)**	−17.1 ± 13.9	26	−45	−9.52 ± 12.2	20	−29	0.581	*p* < 0.001
Circular Radii * (mm^2^)	265.5			555.5				*p* < 0.001

Note: * Only mean model data are available. ** Two-tailed paired observations *t*-test.

## Data Availability

Additional pertinent data are available upon reasonable request.
